# Evaluation of blood glucose level control in type 1 diabetic patients using deep reinforcement learning

**DOI:** 10.1371/journal.pone.0274608

**Published:** 2022-09-13

**Authors:** Phuwadol Viroonluecha, Esteban Egea-Lopez, Jose Santa

**Affiliations:** 1 Universidad Politecnica de Cartagena (UPCT), Department of Information Technologies and Communications, Cartagena, Spain; 2 Universidad Politecnica de Cartagena (UPCT), Department of Electronics, Computer Technology and Projects, Cartagena, Spain; University of Milano Bicocca, ITALY

## Abstract

Diabetes mellitus is a disease associated with abnormally high levels of blood glucose due to a lack of insulin. Combining an insulin pump and continuous glucose monitor with a control algorithm to deliver insulin is an alternative to patient self-management of insulin doses to control blood glucose levels in diabetes mellitus patients. In this work, we propose a closed-loop control for blood glucose levels based on deep reinforcement learning. We describe the initial evaluation of several alternatives conducted on a realistic simulator of the glucoregulatory system and propose a particular implementation strategy based on reducing the frequency of the observations and rewards passed to the agent, and using a simple reward function. We train agents with that strategy for three groups of patient classes, evaluate and compare it with alternative control baselines. Our results show that our method is able to outperform baselines as well as similar recent proposals, by achieving longer periods of safe glycemic state and low risk.

## Introduction

Diabetes mellitus (DM) is a disease associated with abnormally high levels of blood sugar (blood glucose) due to lack of insulin (type 1 diabetes—T1D) or insulin resistance (type 2 diabetes—T2D). In 2019, approximately 463 million adults worldwide were suffering from DM, which is increasing continuously [1]. More than 1.1 million children and adolescents are living with type 1 diabetes. In addition, there are 4.2 million deaths caused by DM.

T1D can produce little or no insulin, so patients must monitor their blood glucose (BG) level and manually administer insulin doses. Excessive insulin doses, may cause *hypoglycemia*, that is, low blood glucose levels, which can lead to short-term complications, such as drowsiness, shakiness, confusion, loss of consciousness, seizure, or even coma or death [2, 3]. On the other hand, too little insulin can result in *hyperglycemia*, that is, high blood glucose levels and can cause long-term chronic diseases, including retinopathy, nephropathy, and neuropathy [3].

A Continuous Glucose Monitor (CGM) is a sensor, usually a wearable device, that provides real-time readings of BG levels. Combining an insulin pump and CGM with a control algorithm to deliver insulin [4] is an alternative to the traditional patient self-management of insulin injection to manage BG levels in DM. Control algorithms can be classified into: (1) open-loop controls, which require patient intervention and/or external information, such as meal or exercise announcement; and (2) closed-loop controls, which do not require the patient intervention to regulate the dosage [5] but some external information can be useful in avoiding rapid BG growth [6]. In this paper we consider a restricted definition of closed-loop controller in which any information that cannot be automatically passed to the controller and requires the intervention of the user is not a closed-loop controller, a point of view shared by similar works [5, 7]. The combination of a CGM, an insulin pump and a closed-loop control algorithm is usually referred to as an Artificial Pancreas (AP). Since the technology for CGMs and insulin pumps is relatively mature, research for AP has been focused in the last years on suitable closed-loop control algorithms [5, 6, 8–10].

Several methods for closed-loop controls can be found in the literature, including proportional integrative derivate (PID) [8, 11, 12] methods, model predictive control (MPC) [9, 13–15] and expert-based approaches [10]. In brief, PIDs, one of the most used solutions [6], use previous BG samples as feedback to determine the insulin needed to drive the desired glucose concentration in human blood. Their main disadvantage is a poor adaptation to meal disturbances [7, 16] and inability to individual treatment. MPC requires a model to predict future glucose concentration using current BG, insulin delivery and meal intake; then the algorithm calculates the appropriate insulin infusion rate by minimizing the difference between estimated glucose concentration from the model and the target glucose concentration on a prediction time window [9]. These methods depend obviously on the quality of the model and are also sensitive to external disruptions such as food intake or physical activity that cannot be accurately modeled [14, 15]. Expert-based approaches implement case-based logic using experience from medical experts to decide when and how much insulin to deliver [10]. The model requires an expert to create, adjust and evaluate the model, which can lead to human errors. Also, existing empirical models of a patient metabolism cannot be applied to these approaches, hence there are no theoretically-based performance guarantees.

Artificial intelligence and machine learning techniques are increasingly being used in medicine and healthcare. Among them, Artificial Neural Networks (ANNs) involve machine learning algorithms that have been already used for diabetes purposes. They need labeled training data from experts to predict blood glucose concentration based on supervised learning and avoid human error. ANNs perform well for short-term prediction [17]. However, improving the prediction of supervised learning approaches implies high volumes of training labeled data and there still remains the problem of designing an appropriate controller from the predicted BG level. Reinforcement Learning (RL) has been suggested as a more promising alternative [5, 7]. In RL, a software agent makes observations and takes actions within an environment and receives rewards from its actions. By appropriately shaping the reward function, the agent can self-learn the desired goal. Their main advantage is that they are model-free, up to some extent since the environment provides the implicit model, and can learn latent disturbances and adapt to them. Nowadays, there are realistic simulators of the glucoregulatory system, even approved by the United States Food and Drug Administration (FDA) [18], that can be used as an environment in an RL framework [19].

Even though different RL approaches have been increasingly proposed and discussed [5, 7, 16], effective training of agents for BG control has proved to be difficult [7, 13, 20, 21]. Several factors may explain the difficulties. First, most of the RL algorithms are designed to approximately solve a Markov Decision Process (MDP) with a fully observable state space [22], but realistic simulators of the glucoregulatory systems cannot be considered fully observable. Therefore, the environment is at most a Partially Observable MDP (POMDP) and, in spite of their ubiquity in many fields, only very few recent algorithms have been developed specifically for POMDPs [23]. Moreover, POMDPs require a mapping from of true environment states to observable variables that have to be defined by the control algorithm designer [5], usually with a high degree of arbitrariness. This may have a serious influence on the learning ability of the agent because a bad choice of observable states makes state changes and associated rewards not directly related to agent actions. For instance, a delayed response to insulin is a realistic feature of the simulator but it is also related to the choice of POMDP state mapping, because a CGM reading (observation) after an insulin injection (action) does not reflect the actual change of state. Second, compared to other learning environments, there is a remarkable number of design alternatives whose influence is not clear and usually require careful trial and evaluation. Those involve the choice of the RL algorithm (agent) and its underlying neural network architecture, the tuning of agent hyperparameters, the selection of an appropriate reward function, and even the design of the action space that may be adapted to patient specific data [7, 21]. For the sake of conciseness, the design choices related to the aforementioned issues are called implementation strategy in the rest of the paper.

From the above discussion, it is clear that different implementation strategies can lead to effective RL-based closed-loop controls. They may result in a viable controller or not, and with widely different performance, so the implementation strategy is subject to further investigation, as it is addressed in this paper. In fact, straightforward implementations, as we discuss and show in the following sections, do not work properly. Therefore, the RL approach to control is not different to the other main approaches to the control problem, PID and MPC, in the sense that it can be considered a generic approach, with many potential different implementations which are proposed and evaluated [14, 15]. In this paper, we evaluate two different reinforcement learning algorithms to control *in silico* blood glucose levels in T1D and compare them with other well-known alternatives, including a PID controller. We describe and discuss our implementation strategy and related problems and compare it with recent proposals using a different implementation strategy [7, 21]. Our work shares with these proposals the initial premise: to train state-of-the-art Deep Reinforcement Learning (DRL) algorithms, such as Soft Actor-Critic (SAC) [24] or Proximal Policy Optimization (PPO) [25], as a T1D BG closed-loop control, but it differs in several aspects of the implementation strategy that we discuss in the paper. Our results show that our strategy can effectively control BG levels, outperforming control baselines in terms of the fraction of time spent in the desired glycaemic state and risk metrics. All the source codes used for evaluation and training as well as the trained agent policies described in this paper are publicly available in our repository (https://github.com/girtel/AIML4Diabetes).

The paper is organized as follows. Next section contains background information needed to better understand the contribution. Afterwards, we describe our implementation strategy and design methodology. Our results are described and compared with baselines in the Results section, and compared with similar proposals in the Discussion section. Finally, we provide concluding remarks.

## Background and related work

### Type 1 diabetes mellitus

Type 1 diabetes (T1D) is an autoimmune disease involving that the pancreatic islets produce little or no insulin. Insulin is an anabolic polypeptide hormone regulating carbohydrate metabolism and normal glucose level in the circulatory system. Pathogenesis of T1D is not yet known [26], but it is often found in family members who have had a history of this disease. T1D is characterized by recurrent or persistent excessive amount of glucose in blood plasma, that is, hyperglycemia, which can be diagnosed by several criteria defined by World Health Organization (WHO) [27]. Patients with this type of diabetes need to use insulin by external means to maintain blood sugar levels, usually by subcutaneous injection. There are four main types of injectable insulin: rapid-acting insulin, short-acting insulin, intermediate-acting insulin, and long-acting insulin. The insulin dose that a patient has to inject is traditionally planned by a physician according to the patient characteristics and clinical history.

### Models and simulation

Currently, it is possible to experiment with virtual patient populations in simulators to determine the optimal insulin dosage before being used in patients [18, 28]. Several simulators exist to research on T1D models, such as TD1_VPP [28], Dosing-RL Gym [29] or UVA/PADOVA Simulator [18]. TD1_VPP is a virtual patient population software which can generate virtual patients following single-hormone and dual-hormone mathematical models, integrating the effects of exercise in the glucoregulatory system. Dosing-RL Gym is based on an expanded version of the Bergman minimal model, which includes meal disturbances [29].

**Fig 1:**
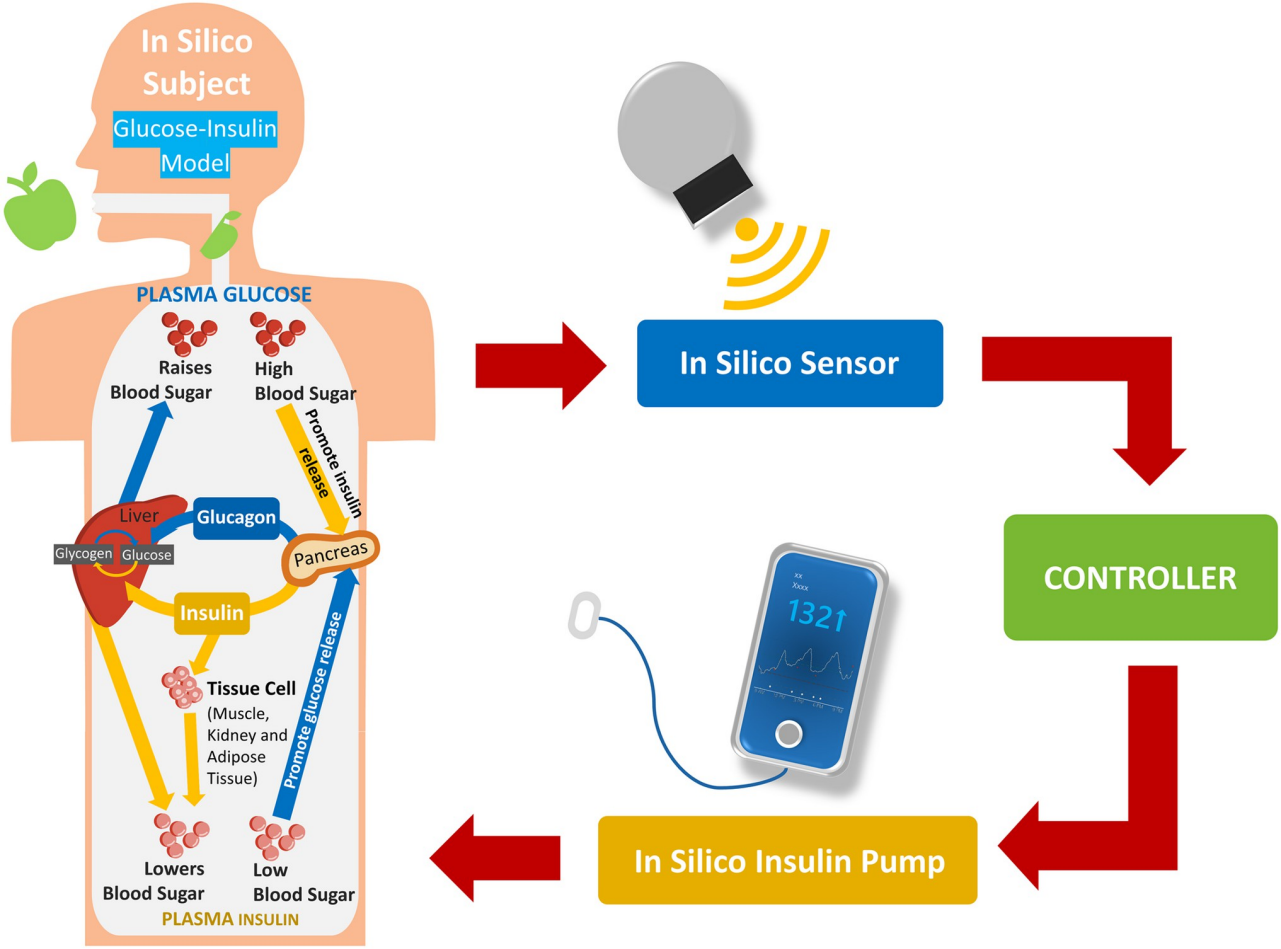
Principal components of the computer simulation environment.

The UVA/PADOVA Type 1 Diabetes Simulator can be used as a substitute for preclinical testing of closed-loop control strategies. The simulator is developed by the Universities of Virginia (UVA) and Padova and has been accepted by the United States Food and Drug Administration (FDA) [18]. It is the most used simulator among *in silico* software, according to [5, 30]. There are four main components of the simulation, which are depicted in Fig 1: (1) *In silico* patient—a model of the glucose-insulin system in a patient; (2) *In silico* sensor—a model of the sensor to measure BG including its error; (3) Controller—a model used to estimate the amount of insulin to maintain blood sugar; and (4) *In silico* pump—a model of discrete insulin delivery and subcutaneous kinetics. In this paper, we use an available open-source Python implementation of the UVA/PADOVA simulator [19], previously used in similar studies [5, 7]. The simulation environment implements the OpenAI gym interface [31], which makes it compatible with several RL frameworks. It also simulates different noisy CGM sensors, insulin pumps and a random meal scheduler.

### Deep reinforcement learning

Reinforcement Learning (RL) refers to a class of methods to optimize the decision process of an *agent* operating on a given *environment*. The decision process is usually considered to be a *Markov Decision Process* (MDP), a discrete-time stochastic control process. Formally, an MDP is defined by a 4-tuple (*S*, *A*, *P*, *R*) of states, *s* ∈ *S*, actions, *a* ∈ *A*, a state-transition function *Pa*(*s*, *s*′) = *Pr*(*s*_*t*+1_ = *s*′|*s*_*t*_ = *s*, *a*_*t*_ = *a*), and reward function *R*_*a*_(*s*, *s*′). The agent is the learner entity that seeks the optimal behavior and is able to perform an action *a*(*s*), which changes the state. In this change of state from *s* to *s*′, the agent obtains a reward *r*, considered as the feedback from the environment. MDP-solving algorithms employ what is called a policy, denoted as *π*, which is a mapping between states and actions; that is *π*: *s* → *a*. Their goal is to reach an optimal policy π*, which maximizes the accumulated sum of rewards over the entire lifespan of the agent. This decision policy can be determined by the state-action function, also called Q-function, *Q*(*s*, *a*), which can be approximated using Deep Neural Networks (DNN). Deep Reinforcement Learning (DRL) refers to algorithms and methods that use DNN to approximate the Q-function or optimal policy.

It is commonly assumed that the MDP has a fully observable state space S, that is, that the agent has access to observations that fully represent the state of the environment. However, the observation may just be a partial representation of the underlying state. A Partially Observable Markov Decision Process (POMDP) is an extension of an MDP, where the agent cannot fully observe the system state. In that case, the MDP 4-tuple is extended with a space of observations, *o* ∈ *O*, and a usually unknown and potentially stochastic function that maps the observations to true underlying states, *T*: *o* → *s*. Partial observability may stem from many factors, including limited sensing capabilities or unknown environment dynamics [23]. Let us remark that the agent with partial observability cannot know which is the real state corresponding to the reward received [32]. Despite the ubiquity of POMDPs in many practical systems [23], most of the DRL algorithms assume an underlying MDP [22, 25]. POMDPs are usually addressed in DRL by augmenting the observation space with the history of past observations and actions and the use of Recurrent Neural Networks (RNN) [23] in the architecture of the learning algorithm. Only recently, a few algorithms have been specifically designed to deal with POMDPs [23]. Moreover, the state transition in some environments is determined not only by agent actions, but also by exogenous stochastic input actions [33]. Efficient methods to deal with this kind of environments are discussed by Mao *et al*. [33]

Most of the state-of-art DRL algorithms used for MDPs are based on actor-critic methods: temporal difference learning algorithms that separate representations of value functions and policies explicitly [22]. The actor selects actions in the action space, while the critic estimates the value function from the action made by the actor. They can be applied to either discrete or continuous action spaces. However, these methods show poor sample efficiency and stability convergence properties. A variety of techniques have been developed to address those problems [22, 25, 34]. In this paper, we have used Soft Actor-Critic (SAC), a widely used continuous-state DRL algorithm [7, 24], whose policy maximizes a trade-off between the expected return and entropy, a measure of randomness in the policy, which ensures higher robustness and stability [22]. Maximum entropy policies have been shown to solve POMDP with unobserved rewards [32]. Besides SAC, we have used Proximal Policy Optimization (PPO), another popular DRL algorithm [25]. PPO ensures that its policy does not change much from the previous policy updates, leading to smooth learning and avoiding variance in training. The tradeoff between SAC and PPO is stability and sample efficiency. PPO tends to be more stable and uses more data, whereas SAC tends to be the opposite. PPO is also claimed to work well on POMDPs [35]. Both allow the use of RNNs in their architecture.

### Related work

State-of-the-art control algorithms for AP systems on devices already on the market are based on PID and MPC approaches [6, 11, 12, 14, 15, 36, 37]. PID and MPC controllers usually work as hybrid closed loop system, requiring announcements of meal carbohydrates amount and exercise activity [37]. Two commercially available FDA-approved systems are Metronics 670G and 770G using PID [36–38] and Tandem Control-IQ using MPC [36, 37]. With PID and MPC, most commercial products avoid hypoglycemia overnight by utilizing Predictive Low Glucose Suspend (PLGS) [37]. PLGS technology predicts glucose concentration trends, then suspends insulin delivery before hypoglycemia occurs. A PID controller is simple but have problems to adapt to meal consumption [6, 7]. Several variations to the basic PID approach have been proposed, as the use of insulin feedback (IF), which improves its performance [11, 12]. MPC controllers are more proactive than PID in insulin delivery by predicting BG levels, but they need a minimal compact mathematical model. Di Ferdinando *et al.* [14] and Borri *et al.* [15] use nonlinear differential difference equation (DDE) models for the endogenous insulin delivery rate (IDR), which is better accounted for in these models. Since the IDR cannot be neglected for T2DM patients, and the DDE model reproduces it accurately, MPC that use DDE usually address T2DM. Their results show that MPC provides good performance as long as a minimal compact model is available. However, as the complexity of the model increases, MPC approaches are not tractable and one has to resort to other control methods.

Among these methods, the number of data-driven models for prediction of BG in T1DM is increasing [30]. Reinforcement learning is being used in recent research works in the field of health care. For instance, the RL Q-Learning algorithm was applied on discrete action space simulation for radiotherapy to understand scenarios of tumor growth and its treatment plan [39]. RL agent-based models have been used on continuous state and action spaces problems to find cytokine therapy for sepsis, reducing mortality from 49% on average to 0.8% [40]. RL is suitable for time sequence problems, as in the glucoregulatory system. Furthermore, the agent can learn the policies without the need for labeled data as in supervised learning methods [5, 16]. The ability of RL to capture food intake patterns without human input makes it a good candidate for a fully closed-loop system and more responsive and safer policies [7].

Particularly about T1DM control, a recent review discusses most of the approaches used so far in this topic [5]. This review exposes the wide variety of alternatives used in almost all the defining elements of the RL framework, such as the definition of the state space, the action space, class of RL algorithms used and the reward function, what we have called the implementation strategies. To mention a few which differ in the class of RL algorithms, in [16] a control based on RL is proposed and the value function is not approximated by a DNN, but by a quadratic function, and in [41] the value function is approximated by a Gaussian process. In both cases, robust solutions are provided, but simplified glucose models are used. On the contrary, in this paper we use a more realistic simulator which generally requires DRL to approximate the value function.

Fox *et al*. adopt an approach similar to the one adopted in this paper in two recent papers [7, 20]. In fact, they share the basic premise with this paper: training a DRL agent for BG closed-loop control. However, they employ different implementation strategies. In their first one [20], the state space comprises the previous 24 hours of CGM samples and insulin doses at 5-minute intervals, but the action space is discrete and made of only three insulin doses. Three relatively simple DNN architectures were used to approximate the value function and the improvements over the PID baseline were not particularly noticeable. In their second work [7], the state space is also made of CGM and insulin samples but only from the four previous hours and the action state is continuous, so the SAC algorithm is used. Additionally, the reward functions differ in both cases. In contrast, in this work we use only the last CGM sample as state but take a relatively long interval of 30 to 60 minutes between observations and actions and define a simpler reward function. A hybrid model-based approach is discussed by Yamagata *et al*. [13], which uses a discrete action space combined with meal announcement. Finally, very recently, Lin *et al*. [21] proposes a combination of machine learning methods for BG control: the controller uses a DRL SAC agent which is driven by a PID control as an initial policy and, in addition, the observation state is extended by the predictions of a dual attention network. Finally, the actions are also regulated by an adaptive safe action. The results of the last three aforementioned methods [7, 13, 21] are further compared with ours in the Discussion section.

## Implementation strategy and methodology

In this section, we describe and discuss the design process and choices for our implementation strategy for a BG closed-loop control based on DRL. Our goal is to balance blood glucose as long as possible with low risks. First, we define the state and action space and discuss the reward function. Afterwards, we conduct an initial evaluation based on naive strategies to determine the features of the environment that may have more influence on the agent learning. This leads us to refine our design and propose an implementation strategy that is evaluated in the Results section.

### Analysis of environment and initial design

The simglucose simulator environment implements the UVA/Padova glucose model [42] and provides CGM sensors that sample the BG level through a noisy (stochastic) process as well as a random and patient-dependent meal schedule. From this description, it is clear that the environment should be considered a POMDP, since the CGM observations of BG levels include noise reads from sensors. In fact, the underlying hidden states of the environment, *s*, are given by the glucose model states [42], rather than the BG level. Moreover, the dynamics of the environment, that is, the state transitions, are not only determined by the actions taken by the agent (insulin dose injected), but also by exogenous stochastic input actions [33], such as the intake of CHO (meals) or physical exercise. However, since simglucose does not consider physical exercise, unlike T1D_VPP [28], we restrict our study to external meals.

All the above considerations have an influence on the DRL controller design. We first define the elements of the DRL algorithm for our problem as follows:

#### Observations and state

The mapping of the observable variables may determine whether the environment is a POMDP or an MDP. As an example, the number of frames included in the observations in the Atari Pong game makes it become a POMDP or a MDP [43]. We start by using only CGM samples as observation variables, since they are readily available. Unlike the work in [7], we do not use past actions (insulin doses) in the observations. Since we target a closed-loop controller, we do not include CHO intake as part of the observation, which has to be announced by the user, even though some devices may facilitate its announcement [44]. We start by using only the current observation, given by the last CGM sample, $o\in {\mathbb{R}}^{+}$. The CGM sample frequency is three minutes per environment step.

#### Action

The action is the amount of basal insulin that the patient gets injected. It is a decimal number, ranging between 0 to 30 units, *a* ∈ [0, 30], according to the specifications of the insulin pumps implemented in the simulator.

#### Goals and risk metrics

Safety is crucial in healthcare applications. The main goal of our BG controller is to balance the BG level for as long as possible with low health risks. A commonly used metric of risk associated with BG levels is the blood glucose risk index (BGRI), and it has also been used to measure the performance of control methods [45]. BGRI is a measure of glucose variability and associated risks and it is based on a symmetrization of the BG measurement scale [45]. The Clarke BGRI is defined as *BGRI* = *LBGI*+ *HBGI*, where *LBGI* and *HBGI* are computed over a series of n CGM samples as:

$$\begin{array}{c} LBGI=\frac{1}{n}\sum_{t=1}^{n}rl\left(BG_{i}\right) \end{array}$$ (1)

and

$$\begin{array}{c} HBGI=\frac{1}{n}\sum_{t=1}^{n}rh\left(BG_{i}\right)) \end{array}$$ (2)

where *LBGI* and *HBGI* represent the risk associated with low and high BG levels. They are computed from the following function:

$$\begin{array}{c} f\left(BG\right)=1.509\times [log{\left(BG\right)}^{1.084}-5.381] \end{array}$$ (3)

Noted that BG is measured in mg/dL. *f*(*BG*) is the basis to calculate the BG risk function using the formula *r*(*BG*) = 10 × *f*(*BG*)^2^ and separating it as low *rl* and high *rh* as follows:

$$\begin{array}{c} rl\left(BG\right)=\{\begin{array}{cc} r(BG), & \text{if}\;f(BG)<0.\\ 0, & \text{otherwise}. \end{array} \end{array}$$ (4)

$$\begin{array}{c} rh\left(BG\right)=\{\begin{array}{cc} r(BG), & \text{if}\;f(BG)>0.\\ 0, & \text{otherwise}. \end{array} \end{array}$$ (5)

Similar risk measures have been defined and used in the literature. For instance, in [7] the Magni risk function is used, defined as:

$$\begin{array}{c} ri\left(BG\right)=10[3.35506{\left({\left(ln\left(BG\right)\right)}^{0.8353}-3.7932\right]}^{2} \end{array}$$ (6)

**Fig 2:**
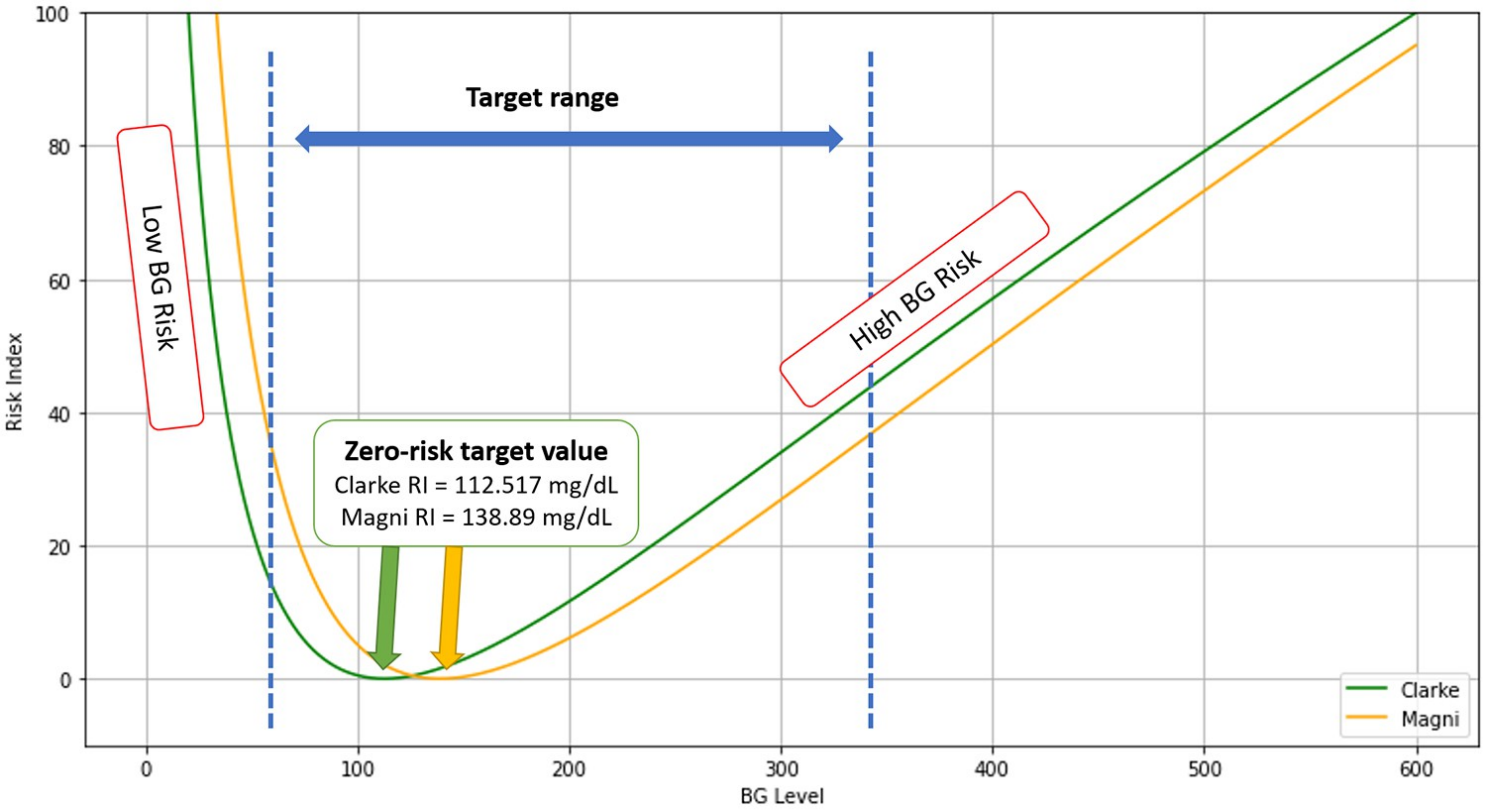
Blood glucose risk index function. Our target value is BG level at 112.517 mg/dL for Clarke RI and 138.89 mg/dL for Magni RI and having a zero BGRI implies that there is no risk for a patient at this point (Zero-risk). This plot shows graphically this point, including the left and right sides of the zero-risk target value, i.e., LGBI and HBGI, respectively. BG level at 70-350 mg/dL is considered as the target range.

The curves from Clarke and Magni are shown in Fig 2. As can be seen, the BGRI adequately captures the increased risk associated with hypoglycemia for the patients.

It is common practice to define the reward function in terms of the Clarke or Magni RI [7, 20]. But, as we discuss next, they may not be adequate to capture our intended goal.

#### Termination limits and safety

In DRL, an environment finishes when some condition is met. For instance, in standard OpenAI gyms such as the BipedalWalker [46] the episode finishes when the robot falls. In simglucose, the episode ends when the BG level goes out of a predefined range. T1D patients should aim for a target range of 70–180 mg/dL [47]. We have configured simglucose to end the episode when BG < 70 mg/dL or BG > 350 mg/dL in order to try to avoid dangerous BG levels. Let us note that this is a quite conservative range. In contrast, in [7], episodes are done when the BG falls below 10 mg/dL or raises over 1000 mg/dL.

#### Reward

A crucial, and problematic, aspect of DRL is the need to design a reward function that helps the agent learn the intended goal [43]. In our solution, we have tried different approaches. In particular, we have tried using the negative of the Clarke BGRI as reward function, in order to keep the BG at the desired level, but in this case negative rewards induce the agent to terminate early, leading to dangerous regimes. Basically, the agent learns to inject more insulin to avoid keeping receiving negative rewards, provoking hypoglycemia. A termination penalty to correct for this behavior is usually introduced, as is done in [7]. From our point of view, this solution is not satisfactory, because the value of the reward at termination becomes effectively another hyperparameter. It requires to be tuned for the expected duration of the episodes. In fact, as an extreme case, since the desired goal is to avoid termination at all, the value should be set to infinity, or at least to a value high enough to counteract the expected lifetime of the patient. We have tried a different strategy: combined with the conservative termination limits mentioned above, we use a simple reward to encourage large episodes, which results in extended periods within adequate BG level regimes. Of course, our reward function can be refined, for instance, considering more sophisticated safe zones. Therefore, the reward is simply:

$$\begin{array}{c} reward=\{\begin{array}{cc} 1, & \text{if}\;BG\in [70,350]\;\text{mg/dL}.\\ 0, & \text{if}\;BG\in [10,69]\;\text{or}\;\left[351,1000\right]\;\text{mg/dL}.\\ -100, & \text{otherwise}. \end{array} \end{array}$$ (7)

#### Discount factor

The discount factor helps to balance the importance of immediate and future rewards. Since the effect of insulin on the BG is usually delayed, we set it to a relatively large value of *γ* = 0.999.

#### Virtual population

The UVA/PADOVA simulator provides parameters for fully specifying the glucoregulatory system of patients in three groups: children, adolescents, and adults, each category including 10 patients. According to the *simglucose* developer [48], the patient parameters correspond to the 30-patient subset available for the academic edition of the 2008 commercial UVA/PADOVA simulator. The commercial version provides a virtual population of 100 patients in each group.

Although 30 patients cannot cover all the possible variations in a heterogeneous population, the size of our population is similar to most of the previous works based on RL: according to the systematic review by Tejedor [5], only 3 out of 23 proposals that used *in silico* patients have over 30 patients. These proposals use 100-patient sets from the UVA/PADOVA simulator. Moreover, ten of the reviewed proposals use just one *in silico* patient. Unlike other methods, such as model-based classical control (MPC) [14, 15], which have an almost negligible computational cost and can be evaluated on thousands of virtual patients, methods based on RL typically use a reduced number of patients. There are two main practical reasons behind these low numbers. First, training RL agents is a highly time-consuming and resource-intensive task. Effective training of a single patient with a set of parameters and hyper-parameters typically takes seven to ten days with our mid-level computing facilities (intel i9-10920X, 64 GB RAM, 2 Nvidia RTX 2080 GPUs). We are able to train 4 to 8 patients in parallel. Second, even if enough time and computing power is available, to train an RL agent, we have to rely on a proven training environment and a set of validated virtual patients. Generating additional patients is possible by sampling from the joint distribution of the model parameters, as described in [9], and variations of this generation method have been used by Di Ferdinando [14] and Borri [15]. However, the values of several parameters were not published, which makes it necessary to guess some of them. Pompa *et al* have very recently compiled the required parameters for future implementations [49]. Nevertheless, we consider that the effort required to rigorously generate patients is beyond the scope of the current paper.

Thus, for an initial search for a viable implementation strategy for RL, which is the paper goal, we consider that 30 patients split into age groups is a reasonable trade-off. As said, our choice is in line with most of the previous works on this topic and even surpasses most of them. Once a viable implementation strategy has been established, a more comprehensive training campaign can be carried out, including the generation of additional virtual patients.

### Initial evaluation

We conducted a series of initial tests to determine the features of the environment that may have a greater influence on the agent learning, according to the initial choices described in the previous section. Simglucose comes with a population of 30 virtual patients: 10 adolescents, 10 adults, and 10 children, which statistically represent different cohorts of patients [19]. We have tested on one patient from each group the initial alternatives that are summarized below and in Table 1.

**Table 1:** Summary of initial evaluation. All columns show average ± 95% confidence intervals.

Subject	Alg/Obs/Reward	Length(min)	Hypoglycemic(%)	Hyperglycemic(%)	Euglycemic(%)
Child#1	PPO/O1/R1	234 ± 50.921	0.0077 ± 0.003	0.44 ± 0.03	0.550 ± 0.040
Child#1	PPO/O2/R1	213.3 ± 34.836	0.004 ± 0.004	0.41 ± 0.06	0.58 ± 0.069
Child#1	PPO/O1/R2	42.9 ± 9.8369	0.074 ± 0.009	0± 0	0.925 ± 0.0093
Child#1	PPO/O2/R2	41.1 ± 6.7217	0.075 ± 0.007	0 ± 0	0.92 ± 0.0079
Adolescent#1	PPO/O1/R1	617.7 ± 155.75	± 0	0.48 ± 0.11	0.510 ± 0.11
Adolescent#1	PPO/O2/R1	536.7 ± 147.89	± 0	0.58 ± 0.09	0.415 ± 0.094
Adolescent#1	PPO/O1/R2	59.4 ± 1.8709	0.050 ± 0.001	0± 0	0.94 ± 0.0015
Adolescent#1	PPO/O2/R2	60 ± 0	0.05 ± 0	0 ± 0	0.95 ± 0
Adult#1	PPO/O1/R1	555.6 ± 157.16	0 ± 0	0.57 ± 0.14	0.42 ± 0.1
Adult#1	PPO/O2/R1	505.5 ± 121.09	0.0057 ± 0.001	0.41 ± 0.08	0.57 ± 0.08
Adult#1	PPO/O1/R2	63.6 ± 0.85843	0.047 ± 0.000619	0± 0	0.95 ± 0.0006
Adult#1	PPO/O2/R2	63.6 ± 0.85843	0.047 ± 0.000619	0± 0	0.95 ± 0.0006
Child#1	SAC/O1/R1	39 ± 0	0.0769 ± 9.9276e-18	0 ± 0	0.92308± 0
Child#1	SAC/O2/R1	63.6 ± 19.166	0.05248± 0.010184	0.030 ± 0.064	0.91773± 0.05
Child#1	SAC/O1/R2	36.3 ± 0.64	0.082± 0.001	0 ± 0	0.91731± 0.001
Child#1	SAC/O2/R2	36.9 ± 0.98	0.08± 0.002	0 ± 0	0.91859± 0.002
Adolescent#1	SAC/O1/R1	123.6 ± 12.13	0.02± 0.001	0 ± 0	0.97538± 0.001
Adolescent#1	SAC/O2/R1	97.2 ± 15.30	0.03± 0.0046488	0 ± 0	0.96773± 0.004
Adolescent#1	SAC/O1/R2	54 ± 0	0.05± 4.9638e-18	0 ± 0	0.94444± 0
Adolescent#1	SAC/O2/R2	64.5 ± 7.57	0.04± 0.0044488	0 ± 0	0.95248± 0.004
Adult#1	SAC/O1/R1	87.9 ± 14.83	0.03± 0.003685	0 ± 0	0.96466± 0.00
Adult#1	SAC/O2/R1	137.1 ± 29.87	0.02± 0.005159	0 ± 0	0.97598± 0.00
Adult#1	SAC/O1/R2	59.1 ± 0.98	0.05± 0.00086268	0 ± 0	0.94921± 0.0008
Adult#1	SAC/O2/R2	66.6 ± 2.68	0.04± 0.0018355	0 ± 0	0.95481± 0.001

- Algorithms: **PPO** and **SAC**, with a standard configuration using dense DNN with two hidden layers of 256 units. In addition, we used an alternative recurrent architecture intended to capture temporal context, which uses as actor and critic networks a RNN with a 10 LSTM cells. We call this variant **PPO-RNN** and **SAC-RNN**. The performance of PPO-RNN and SAC-RNN was similar to the one shown in Table 1 and will not be reproduced.
- Observation space: we used as observation both **(O1)** the current CGM sample and a **(O2)** vector of the past 20 CGM samples, corresponding to the previous hour at 3-minute intervals.
- Reward functions: we used **(R1)** the one point per step reward of Eq (7) and **(R2)** the negative of the Clarke BGRI with a termination penalty. In all the case we set the termination limits to BG<70 or BG>350.
- Meal schedule: the simulator selects a non-deterministic meal schedule particular to each patient according to the Harris-Benedict algorithm [7].

We used the PPO and SAC implementations from *stable-baselines3* [50] and PPO-RNN, SAC-RNN from *TensorFlow Agents* (TF-Agents [51]) and trained it on two Nvidia GeForce RTX 2080 GPUs and Intel Core i9-10920X CPU @ 3.50GHz 12 cores. All agents were trained for 1 million steps, keeping the best model (best average reward) and with a maximum episode length of 10 000 steps (a step represents 3-minute interval).

From the average length of the episode, it can be seen that the agents are not able to keep a safe control of BG levels beyond 10 hours. In general, PPO is able to achieve longer duration within a safe BG range, because it injects lower insulin levels and patients spend more time in a hyperglycemic state. In fact, most of the episodes with PPO end because of high BG levels. On the contrary, SAC tends to inject insulin aggressively and patients go rapidly to a hypoglycemic state, ending the episode. In both cases, using just the current CGM observation (O1) or the last one-hour interval of CGM observations (O2) has little influence, and using the simple reward (R1) works better than the negative of the Clarke BGRI.

### Discussion and refinement of design

From the previous evaluation, we hypothesize that the three main characteristics of the simulation that affect the lack of training success are: (1) The action drives the BG level to lower values, but the rise of BG level depends on the environment dynamics and as consequence of the exogenous input actions, that is, the intake of CHO in the meals. (2) The observation (CGM) is a noisy sample of the BG level. (3) The effect of actions on the BG level is delayed. That is, applying an action does not immediately decrease the BG level. Even though those effects are expected from the initial analysis of the environment, and not particularly surprising, we think they deserve further discussion.

Regarding (1), the agent should learn the policy to deal with this fact, that is, that it does not need to deliver insulin when the BG level is low. In fact, our results show that the PPO agent is able to learn policies that anticipate the meal consumption and the subsequent rise of BG. However, they are not enough precise to control adequately the BG levels. This is probably because of the conservative BG range that leads to an early episode ending. Regarding (2), let us just notice that the reward is usually assigned according to the actual BG level, not the CGM sample, which seems to negatively affect learning since it makes termination penalties appear random. But even using only BG levels, instead of noisy CGM samples, did not improve learning. Regarding (3), both the large discount factor and the recurrent architecture should have improved learning. But the termination limits of the environment, the glucose dynamics and the randomness of meal schedules make it hard to learn:if the agent tries high insulin does, the patient goes very quickly to hypoglycemia, and the episode ends, and when the agent injects low doses, meals, which are not included in the observation, raise the BG level ending also the episode. Therefore, it seems that the combination of all these factors prevents the agent from properly learning. Both (2) and (3) stem from the fact that we are dealing with a POMDP and the recurrent architecture should improve learning, but several variations tried in our tests did not actually improve much. We could have tried changing the RNN architecture and other hyperparameters, but due to the large parameter space, we chose to focus on the delay of actions as follows.

#### Observation frequency and insulin response

We just configured the environment to decrement the frequency of the observations and actions, instead of using the usual 3 or 5-minute CGM sample resolution. Even though the samples are taken, since the simglucose environment is running and updating the state in *mini-steps* of 1 min, they are passed less frequently to the agent, which then provides an action. The rationale is simple: to let the agent observe the actual effects of its actions, that is, the actual patient insulin response according to the glucose dynamics, instead of a seemingly random transition. Even though it is similar to using as state a history of the previous CGM data, as is done in [7], it improves the learning process and leads to more adequate insulin regimes in our results. Therefore, we have introduced an additional hyperparameter, *observation frequency*, which is actually already present in the simulator (CGM sample resolution) although usually left at the default device value (a CGM sample every 3 minutes) [7]. Let us notice that this new hyperparameter does not increase the complexity compared to using a history of previous samples [7], since in that case, the lengths of the history vectors are also additional hyperparameters to be tuned.

**Fig 3:**
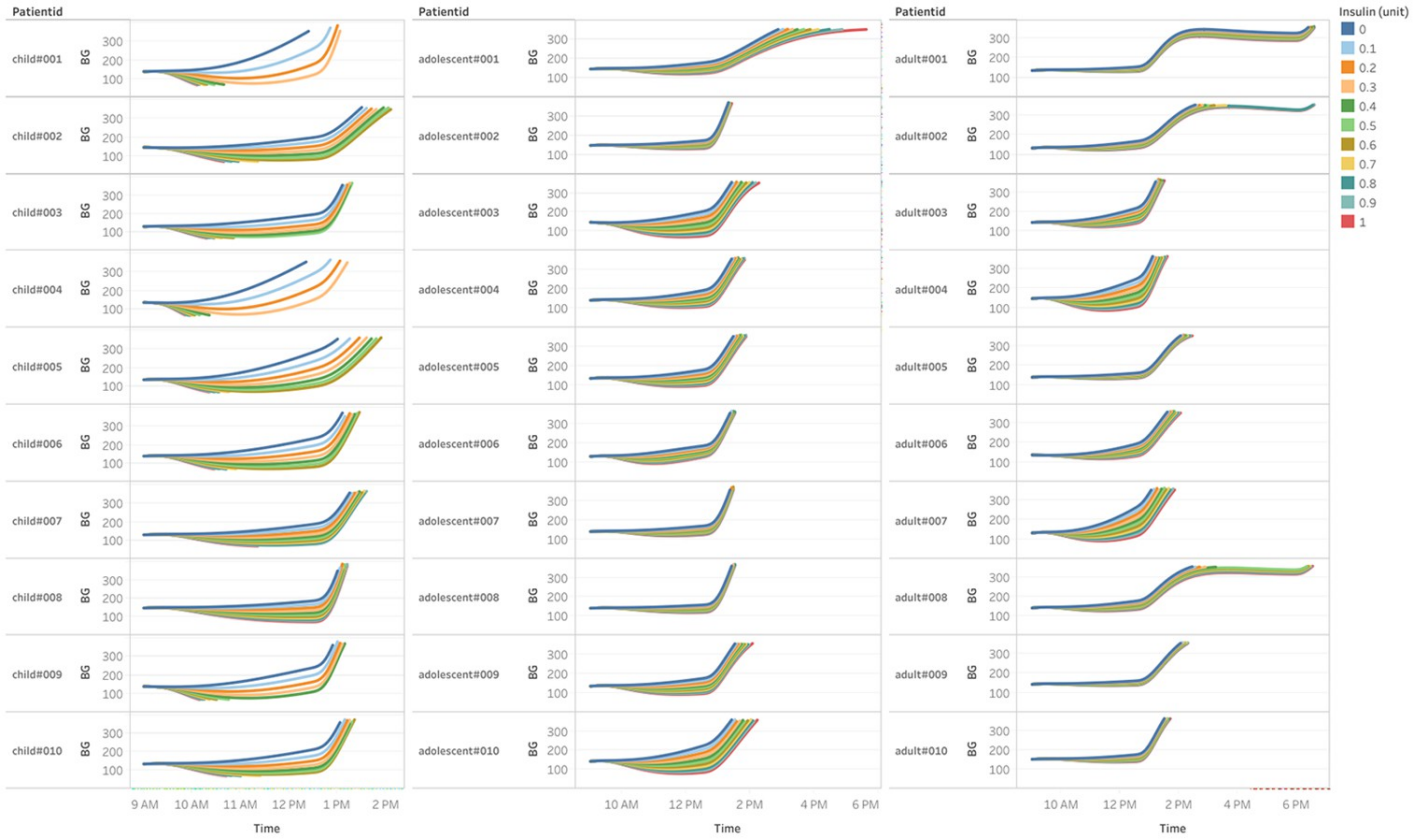
Insulin response time for each patient. The insulin dose depicted in the color legend on the right was injected at 9:00 AM and no meal is taken subsequently.

We selected the frequency for the observations by testing the insulin response time when injecting a given unit of insulin without taking any meals. This delay is different depending on the patient group, as expected, and it is shown in Fig 3: ten different amounts of insulin doses were used to estimate each subject insulin reaction, from 1 to 30 units. As can be seen, the adult response to insulin tends to be more stable and less pronounced and the reduction in BG starts to show around 45 minutes after injection. Adolescent reaction is slightly slower and more pronounced. Finally, children clearly react faster and more strongly to insulin. In fact, high insulin doses quickly drive some of the patients to hypoglycemia and episode termination. From these observations we chose reducing the frequency of observations as follows: for adults, observations are made every 45 minutes (corresponding to 15 three-minute environment steps), adolescent frequency is set to 30 minutes (10 steps) and children is set to 15 minutes (5 steps).

Then, we started over the training of the **SAC**, **SAC-RNN**, **PPO** and **PPO-RNN** agents. After running several experiments, only **PPO-RNN** could learn effective policies. Therefore, we have selected this algorithm and architecture for a full evaluation of performance in the Results section.

### Summary of implementation strategy

We provide here a brief summary of our implementation strategy, before conducting an evaluation and comparison of alternatives in the next section.

From the discussion in the previous section we derive the following implementation strategy: (1) we reduce the *observation frequency* of the environment state (CGM) and, hence, rewards passed to the agent, depending on the subject (45 minutes, 1 hour and 15 minutes for adults, adolescents and children, respectively), (2) we set broad termination limits for the episodes, *BG* ∈ [10, 1000], to let the agent explore more thoroughly the environment; and (3) we use the simple reward function of Eq (8) below, to force the agent to learn to keep the patients in euglycemia for as long as possible.

$$\begin{array}{c} reward=\{\begin{array}{cc} 1, & \text{if}\;BG\in [70,180]\;\text{mg/dL}.\\ 0, & \text{if}\;BG\in [10,69]\;\text{or}\;\left[181,1000\right]\;\text{mg/dL}.\\ -100, & \text{otherwise}. \end{array} \end{array}$$ (8)

Let us note that the reward is accumulated during all the simulation *ministeps* and then passed to the agent. For example, if we set the observation frequency to 10, the environment is going to simulate 10 ministeps before passing the sample to the agent, and, if BG level has been in the desired range all those ministeps, the accumulated reward passed will be 10.

## Results

### Experimental setup

#### Training and evaluation

Our goal is to keep the patient BG level in the selected range for as long as possible. We have trained a PPO-RNN agent for each of the patients with the implementation strategy summarized in the previous section and the hyperparameters listed in Table 2. During the training, there are periods of instability, where the average reward drops, as reported in other studies [7]. Rigorous convergence of the RL algorithms tested in this paper, SAC and PPO, including their variants with RNN, has not been proved analytically, in general, to the best of our knowledge. Only recently, the convergence of PPO to a local minimum of the associated losses has been proved [52]. In practice, the convergence of the algorithms is assumed when the learning curve does not improve over time. In our case, both PPO and SAC tend to show an oscillatory behavior in the learning curves so that the learning curve increases and then drops. PPO is able to recover from this behavior and we stop the training process when the learning curve has stabilized. The reason for these oscillations is likely the presence of unbounded exogenous stochastic inputs, that is, the meals or the noisy observations. We save the policy every 100 training steps and select the policy with a highest average reward as a trained agent. Once trained, the agents are evaluated 20 times with different seeds for all the patients, and statistics for episode length, fraction of time in glycemia states (eu, hypo and hyper) and other metrics are collected. For evaluation, we also set the environment termination limits to *BG* = 10 and *BG* = 1000, in order to test the fraction of time that the patients spend in the different states and make them comparable to similar proposals [7]. Let us remark that patients whose BG reaches levels below or above those limits are considered events that result in serious damage or death.

**Table 2:** Hyperparameters for PPO-RNN.

Hyperparameter name	Value
actor_fc_layers	200, 100
value_fc_layers	200, 100
actor_lstm_size	128, 128
critic_lstm_size	128, 128
num_environment_steps	25000000
collect_episodes_per_iteration	10
num_parallel_environments	30
replay_buffer_capacity	1001
num_epochs	25
learning_rate	1e-3
num_eval_episodes	20

### Baselines

We have compared our results with four baselines: a basal-bolus regime (BB), that simulates the usual self-managed treatment for patients with both T1D and T2D, basal-bolus with cooldown, a PID, and PID with insulin feedback baselines.

#### Basal-bolus Baseline (BB)

A multiple daily injection therapy which involves using long-acting insulin with a dosage of basal:

$$\begin{array}{c} basal=\frac{u2ss\times body\;weight(kg)}{6000(U/min)} \end{array}$$ (9)

where *u*2*ss* is the steady state insulin rate per kilogram *pmol*/*L* × *kg*; and short or rapid-acting insulin (bolus) to regulate blood glucose concentration with bolus:

$$\begin{array}{c} bolus=\left(CHO>0\right)*\left(\frac{CHO}{CR}+\frac{BG_{current}-BG_{target}}{CF}\right)/t \end{array}$$ (10)

where CF is a correction factor, t is the time between samples and CR is a carbohydrate ratio [7].

To obtain a more stable regime, an alternative is to apply a cooldown signal to the basal-bolus insulin delivery policy (**BB-CD**) to ensure that each meal is corrected only once:

$$\begin{array}{c} bolus=\left(CHO>0\right)*\left(\frac{CHO}{CR}+cooldown*\frac{BG_{current}-BG_{target}}{CF}\right)/t \end{array}$$ (11)

where cooldown is 1, if the patient has not had meals in the past three hours and otherwise is 0. Let us note that this treatment requires the patient to be aware of the meal intake and so it is not a closed-loop control. The controls that require explicit knowledge of meal intake are usually called *controls with meal announcement* in the literature.

#### PID baseline (PID)

A closed-loop control which uses a discrete PID controller aims to set the system output to a given target, *s*_*t*_, by setting the control variable *a*_*k*_ as a linear combination of three terms:

$$\begin{array}{c} a_{k}=K_{p}P\left(s_{k}\right)+K_{i}I\left(s_{k}\right)+K_{d}D\left(s_{k}\right) \end{array}$$ (12)

where *P*(*s*_*k*_) = *s*_*k*_ − *s*_*t*_, $I(s_{k})=\sum_{i=0}^{k}(s_{i}-s_{t})$ and *D*(*s*_*k*_) = |*s*_*k*_ − *s*_*k*−1_|.

We use the optimal values for the PID parameters, *K*_*p*_, *K*_*d*_, *K*_*i*_, for each patient provided by Fox *et al.* [7]. In fact, insulin in blood suppresses the next insulin production, called insulin feedback. Thus, we introduced PID control with insulin feedback (**PID-IF**) based on [11],

$$\begin{array}{c} a_{d}\left(k\right)=\left(1+\gamma /K_{pi}\right)*a_{k}-\gamma *I_{p}\left(t\right) \end{array}$$ (13)

where *γ* is the degree of suppressed insulin delivery by the current plasma insulin, which is equal 0.5, *K*_*pi*_ is the normalized insulin concentration in units. *K*_*pi*_ is equal to 1, and *I*_*p*_(*t*) is the model of pharmacokinetics of insulin adapted from [12], given by

$$\begin{array}{c} I_{p}\left(t\right)=\frac{I_{B}}{K_{pi}\left(\tau_{2}-\tau_{1}\right)}\left(e^{-t/\tau_{2}}-e^{-t/\tau_{1}}\right) \end{array}$$ (14)

where the parameter *I*_*B*_ is the insulin injected in the previous action, and *τ*_1_ and *τ*_2_ are time constants (in minute) associated with the subcutaneous absorption of insulin equal to 55 and 70, respectively.

### Episode length

First, we examine the average episode length in evaluation. Table 3 shows the average fraction of episodes that were completed by each patient group. Note that an episode is terminated when the BG level goes out of the 10-1000 mg/dL range, which means that the patient has reached a BG level that may result in serious damage or death. A primary goal, therefore, of any control method is to avoid early episode termination. Almost all control methods are able to make all the patients finish the 10-day evaluation simulations for all the groups except for BB. The adult group is the easier to control and all patients. PPO-RNN, BB-CD, PID and PID-IF were able to finish the 10-day evaluation period for all the 20 simulations. On the contrary, the children are the most difficult group and BB can only reach on average 78% of full episodes, that is 7.8 days. The introduction of a cooldown (BB-CD) improves basal-bolus overall episode length. But being able to finish the evaluation period is not enough to determine the quality of the treatment: BG levels of patients must be kept in the desired range for as long as possible. In the following sections, we examine how the controls keep the state of the patient during that period.

**Table 3:** Fraction of completed 10-day evaluation reached for each method and group.

Group-Method	Fraction of full episode (average % ± 0.95 CI)
Children-BB	77.6 ± 4.7
Children-BB-CD	100 ± 0
Children-PID	100 ± 0
Children-PID-IF	100 ± 0
Children-PPO-RNN	100 ± 0
Adolescent-BB	88.2 ± 3.8
Adolescent-BB-CD	100 ± 0
Adolescent-PID	100 ± 0
Adolescent-PID-IF	100 ± 0
Adolescent-PPO-RNN	100 ± 0
Adult-BB	91.9 ± 3.3
Adult-BB-CD	100 ± 0
Adult-PID	100 ± 0
Adult-PID-IF	100 ± 0
Adult-PPO-RNN	100 ± 0

### Risk index and glycemic states

**Fig 4:**
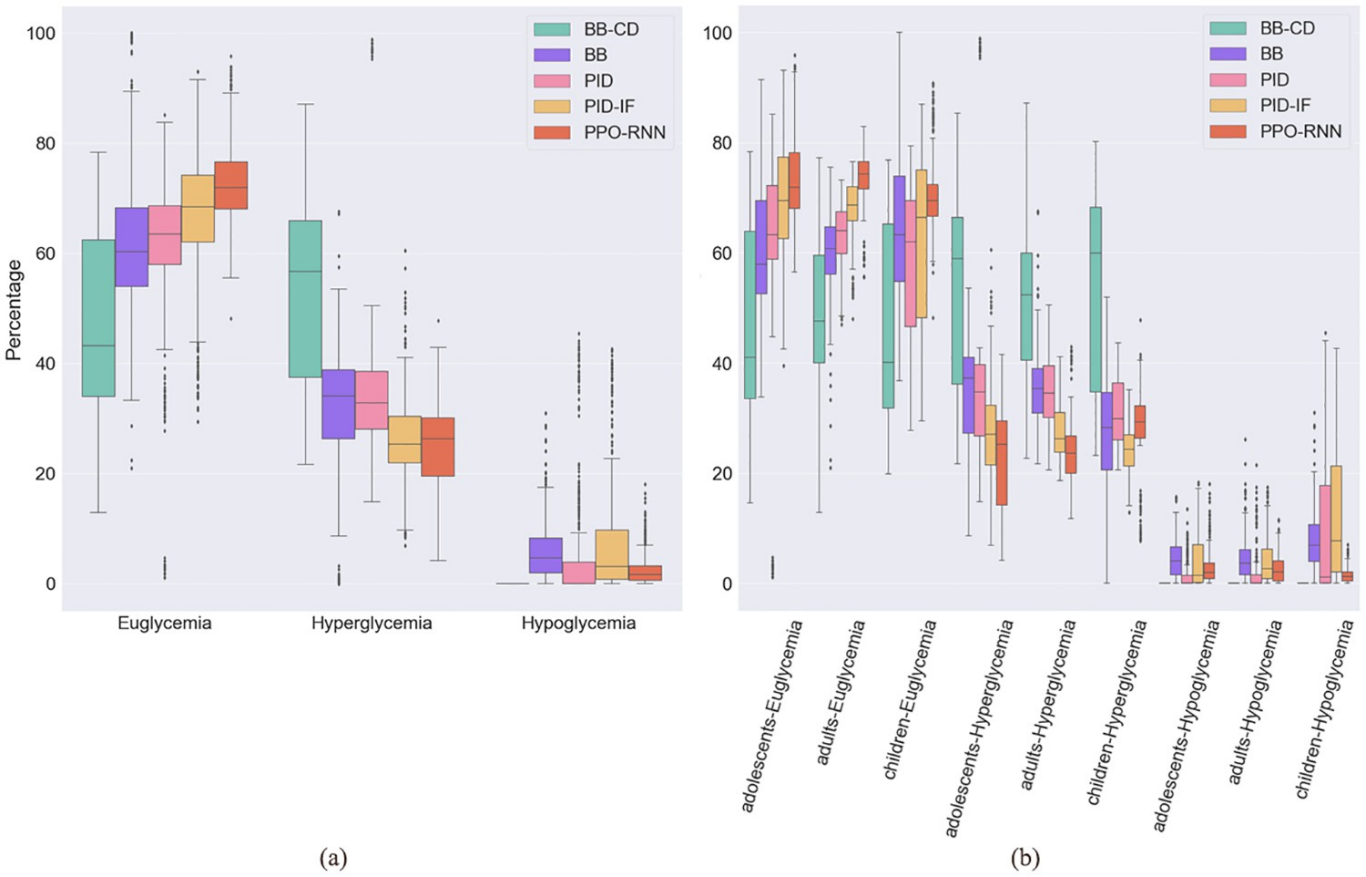
Comparative fraction of time spent in different glycemic states. (a) Global glycemic state (b) Glycemic state by group.

We compare the results of BB, BB-CD, PID, PID-IF and PPO-RNN controllers for the risk index and fraction of time spent in hyper, hypo and euglycemia. The aim is to determine how well controllers regulate the risk of hypoglycemia and hyperglycemia. First, Fig 4a shows with boxplots the distribution for all the methods evaluated. As can be seen, PPO-RNN makes all patients spend more time in an euglycemic state than the baselines, which is the actual goal of this mechanism. Both the median and 25 and 50 percentiles are above those of the other methods. In addition, PPO-RNN also outperforms the baselines globally in terms of the fraction of time spent in hyperglycemia and hypoglycemia. It is instructive to remark how the distributions are more informative in this case that single point estimates, such as the median or mean. For instance, even though the median for the hypoglycemic fraction is similar for PPO-RNN and PID, we can see that a remarkable number of patients spent an unacceptably large fraction of time in hypoglycemia with all PID variants.

In Fig 4b we show that PPO-RNN also outperforms the baselines when the patient groups are examined separately. The results for children are especially noteworthy, since, as we have discussed, it is the most difficult group to train. For that group, we remark that: (1) PPO is able to perform especially well to avoid hypoglycemia, unlike PID, which fails clearly in this aspect; and (2) BB seems to provide reasonable results for children. However, for BB, the results of the previous section have to be taken into account, that is, that the control is only able to reach 78% of the full episode on average. In other words, the control may provide a good response on average for typical BG levels and meal intakes, but not be able to react to unusual variations, which drive the patient to a dangerous state. On the other hand, BB-CD is able to practically eliminate hypoglycemia, but at the cost of a much higher hyperglycemia for all groups.

**Fig 5:**
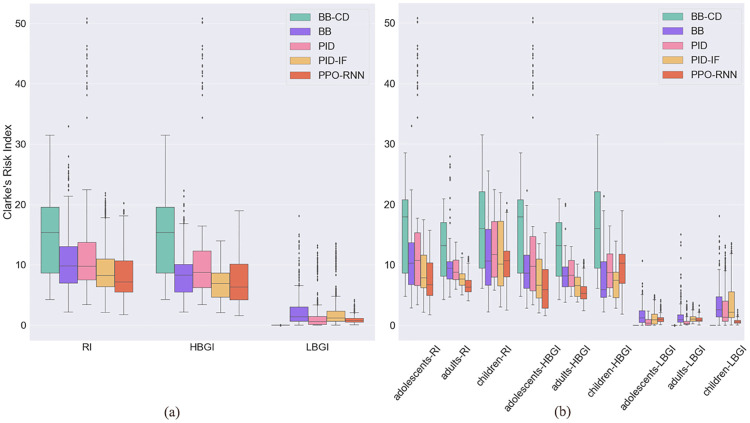
Comparative fraction of risk indexes. (a) Global risk index (b) Risk index by group.

Therefore, to better assess the results, it is necessary to look also at the risk index, which informs us whether the patient is at a safe level within the desirable range. For instance, a patient may spend a large fraction of the time in the euglycemic range but with BG levels very close to the hyper or hypoglycemia thresholds, which may make it vulnerable to unusual conditions, such as irregular meal intakes. Recall that RI penalizes more hypoglycemia, because even though both hypoglycemia or hyperglycemia can lead to fatal outcomes, the short-term effects of hypoglycemia can cause T1D patients to have an immediate crisis [37], as opposed to hyperglycemia, whose effects manifest in the long term. We show the average RI, HBGI and LBGI all over the evaluation period globally in Fig 5a and by groups in Fig 5b. In both cases, PPO-RNN outperforms the other controls and keeps all the RI metrics within reasonable levels, unlike the baselines, especially PID and BB-CD, which show high RI metrics. These results show that PPO-RNN keeps the patients within safe limits most of the time, unlike the baselines, which do not success in smoothly control BG levels: Even though the patients may be euglycemic, they exhibit less safe BG levels. This is the reason why, combined with poor adaptability, some BB-controlled patients are not able to finish the 10-day episodes.

## Discussion

The Results section shows that our implementation strategy can effectively control BG levels and consistently outperforms the baselines. A first result to remark is that our tests showed that no patients controlled by PPO-RNN terminated earlier in any of the evaluation trials. Early terminations are called *catastrophic failures* by Fox [7], since they signal potentially fatal BG levels. Our work in this aspect shows slightly better results than Fox [7], which shows evaluation failures of around 0.1%, but their evaluation extended to 100 10-day replications per patient, whereas ours has been limited to 20 10-day replications. On the other hand, we define a failure when BG goes below 10 mg/dL, whereas they apply an even lower threshold of 5 mg/dL. PPO-RNN clearly improves over Yamagata [13], which reports very high failure percentages, with some patients completely unable to finish any episode. Of course, the critical nature of BG control requires exhaustive evaluation of results. Closed-loop controllers must be evaluated by its capability to keep the BG within an acceptable range for years. As a future work we will evaluate for extended time periods, but, since our tests show that patients keep BG levels above 50 mg/dL and below 400 mg/DL in all the replications so far, we are confident PPO-RNN can avoid failures for longer episodes.

Attending to the time spent in euglycemia, our results are in line with the ones reported by Fox [7], but outperforms those of Lim [21] and Yamagata [13]. Regarding the former, the implementation strategy, as well as ours, is able to keep euglycemia over 73% of time globally, and, in our case, also for all groups. Our implementation strategy can be considered simpler than the Fox one because our observation space is unidimensional (last CGM sample), which makes training more efficient, whereas Fox uses 96 dimensions (previous 48 CGM and insulin data samples). The extended observation frequency for each patient group is an additional hyperparameter to be optimized in our case, but the choice of 48 previous samples is also a hyperparamter in their case. Lim and Yamagata papers show only marginal improvements over the baselines using different strategies, and in both cases only report 64% of time in euglycemia. Moreover, in all the discussed papers, the children group shows more difficulties to be appropriately controlled with a closed-loop controller. In fact, in the work by Lim [21] the children group is not evaluated at all, despite using a fairly sophisticated control involving DRL driven by PID as initial policy. Similarly, in [13] only three patients of each group were evaluated.

The evaluation discussed in this proposal and the aforementioned works show that the application of DRL to BG control is a challenging task. Our results show, however, that DRL has a great potential as a closed-loop controller. Proposals put forward so far discuss just a tiny fraction of the space of potential implementation strategies for DRL-based BG control. The fact that the employed ones at the moment are relatively simple but exhibit a performance better than PID and PID-IF, encourages further exploration of this approach. In our case, there is a great margin for improvement. As a future work, we plan to optimize the hyperparameters of our method and test variations in the RNN architecture. We think that changes in the reward function, narrowing the desired range to obtain reward, may help improve the euglycemia fraction. The introduction of an extended observation frequency in our case has been the key to make agents learn effectively. It is introduced as a new hyperparameter, which may be optimized, but we plan to try strategies to make agents learn it. Once the strategy has been optimized, we plan to conduct a thorough analysis of the way the agent applies insulin doses and compare it with those prescribed by clinical practitioners. The goal is to find whether trained policies employ unusual patterns of dosage that may help clinical practice. In any case, we acknowledge a slight limitation of our approach: the small size of the virtual population of patients, which cannot completely reflect a heterogeneous population. As discussed in previous sections, this is a common drawback of current RL proposals, due to demanding time and computation requirements of this method, and specially, the lack of validated patient sets. Since an exhaustive compilation of the required parameters have been recently published [49], we plan to generate and validate additional sets of virtual patients. Finally, as alternative implementation strategies, it should be worth trying algorithms designed specifically to deal with POMDP and input-dependent environments [23, 33].

As a final note, it is important to consider how to practically apply RL-based controls in real patients. Only a proof-of-concept approach can be considered, due to the difficulties for real application. That is, training an agent requires experimentation (insulin injection) on the subject to learn the optimal control, which is out of the question for real patients. It can only be tested on virtual patients and how to transfer it to real patients is a difficult matter [53]. However, our results may be used with more realistic approaches, such as *offline RL* [54]. With this novel method, the agent, usually a neural network with a transformer architecture, is trained on previously collected datasets, without direct experimentation on the subject. Those datasets may correspond to a series of BG levels and insulin doses collected from real patients, but they may also come from simulations on virtual patients, and both can be combined. Surprisingly, agents trained this way may show better performance than the original methods [55, 56], especially if the datasets contain high-reward regions of the state space. Therefore, the availability of methods to generate very diverse datasets for further use as input, combined with additional data sources, is importante in offline RL. The agents proposed here can actually generate such high-rewards datasets.

## Conclusion

In this work, we propose a closed-loop BG level control based on DRL. We discuss the particular characteristics of a realistic simulator of the glucoregulatory system as a training environment for DRL agents and the complexity of their training in this environment. Effective training of such agents can be achieved by very different design choices for the learning process, which we call the implementation strategy.

We describe the initial evaluation of several alternatives conducted on a T1DM UVA/PADOVA simulator and, based on the results, propose a particular implementation strategy based on reducing the frequency of the observations and rewards passed to the agent, and using a simple reward function. PPO-RNN agents are trained with that strategy for three groups of patients, evaluated and compared with PID, PID-IF, BB, BB-CD baselines. Our system is able to outperform common PID and BB strategies in overall terms, attending to healthy glycemic states and the risk index. A critical discussion of the results and a comparison with several recent works is provided, indicating that our system outperforms current solutions at a lower computational cost. Euglycemia is maintained in 73% of the time, and no early termination events (BG out of range) are reported. Hence, our results show DRL as a promising methodology for implementing closed-loop BG control.

There is still large space of potential strategies and margin for improvement in future research. In particular, as a future work, in addition to variations on our current strategy, we consider the use of novel algorithms for POMDP and exogenous stochastic input actions. In our continued work, we plan to consider offline RL and investigate it as a T1D plasma glucose controller.

## References

[1] International Diabetes Federation Diabetes Atlas, 9th edition; 2019. Available from: https://www.diabetesatlas.org.

[2] Bequette BW, Cameron F, Buckingham BA, Maahs DM, Lum J. Overnight Hypoglycemia and Hyperglycemia Mitigation for Individuals with Type 1 Diabetes: How Risks Can Be Reduced. IEEE Control Systems Magazine. 2018;38(1):125–134. doi: 10.1109/MCS.2017.2767119

[3] Khodakaramzadeh S, Batmani Y, Meskin N. Automatic blood glucose control for type 1 diabetes: A trade-off between postprandial hyperglycemia and hypoglycemia. Biomedical Signal Processing and Control. 2019;54:101603. doi: 10.1016/j.bspc.2019.101603

[4] Bekiari E, Kitsios K, Thabit H, Tauschmann M, Athanasiadou E, Karagiannis T, et al. Artificial pancreas treatment for outpatients with type 1 diabetes: systematic review and meta-analysis. BMJ (Clinical research ed). 2018. doi: 10.1136/bmj.k1310 29669716 PMC5902803

[5] Tejedor M, Woldaregay AZ, Godtliebsen F. Reinforcement learning application in diabetes blood glucose control: A systematic review. Artificial Intelligence in Medicine. 2020;104:101836. doi: 10.1016/j.artmed.2020.101836 32499004

[6] Bothe MK, Dickens L, Reichel K, Tellmann A, Ellger B, Westphal M, et al. The use of reinforcement learning algorithms to meet the challenges of an artificial pancreas. Expert Review of Medical Devices. 2013;10(5):661–673. doi: 10.1586/17434440.2013.827515 23972072

[7] Fox I, Lee J, Pop-Busui R, Wiens J. Deep reinforcement learning for closed-loop blood glucose control. In: Machine Learning for Healthcare Conference. PMLR; 2020. p. 508–536.

[8] Renard E, Place J, Cantwell M, Chevassus H, Palerm CC. Closed-loop insulin delivery using a subcutaneous glucose sensor and intraperitoneal insulin delivery: feasibility study testing a new model for the artificial pancreas. Diabetes care. 2010;33(1):121–127. doi: 10.2337/dc09-1080 19846796 PMC2797956

[9] Magni L, Raimondo DM, Bossi L, Man CD, Nicolao GD, Kovatchev B, et al. Model Predictive Control of Type 1 Diabetes: An in Silico Trial. Journal of Diabetes Science and Technology. 2007;1(6):804–812. doi: 10.1177/193229680700100603 19885152 PMC2769684

[10] Ibbini M, Masadeh M. A fuzzy logic based closed-loop control system for blood glucose level regulation in diabetics. Journal of Medical Engineering & Technology. 2005;29(2):64–69. doi: 10.1080/03091900410001709088 15804854

[11] Huyett LM, Dassau E, Zisser HC, Doyle FJ. Design and Evaluation of a Robust PID Controller for a Fully Implantable Artificial Pancreas. Industrial & Engineering Chemistry Research. 2015;54(42):10311–10321. doi: 10.1021/acs.iecr.5b01237 26538805 PMC4627627

[12] Palerm CC. Physiologic insulin delivery with insulin feedback: A control systems perspective. Computer Methods and Programs in Biomedicine. 2011;102(2):130–137. doi: 10.1016/j.cmpb.2010.06.007 20674062

[13] Yamagata T, Ayobi A, O’Kane A, Katz D, Stawarz K, Marshall P, et al. Model-Based Reinforcement Learning for Type 1 Diabetes Blood Glucose Control. In: Singular Problems for Healthcare Workshop at ECAI 2020; Conference date: 29-08-2020 Through 08-09-2020; 2020. p. 1–14.

[14] Ferdinando MD, Pepe P, Gennaro SD, Palumbo P. Sampled-Data Static Output Feedback Control of the Glucose-Insulin System. IFAC-PapersOnLine. 2020;53(2):3626–3631. doi: 10.1016/j.ifacol.2020.12.2044

[15] Borri A, Pola G, Pepe P, Benedetto MDD, Palumbo P. Symbolic Control Design of an Artificial Pancreas for Type-2 Diabetes. IEEE Transactions on Control Systems Technology. 2021; p. 1–16.

[16] Ngo PD, Wei S, Holubová A, Muzik J, Godtliebsen F. Control of Blood Glucose for Type-1 Diabetes by Using Reinforcement Learning with Feedforward Algorithm. Computational and Mathematical Methods in Medicine. 2018;2018:1–8. doi: 10.1155/2018/4091497 30693047 PMC6332998

[17] Robertson G, Lehmann ED, Sandham W, Hamilton D. Blood Glucose Prediction Using Artificial Neural Networks Trained with the AIDA Diabetes Simulator: A Proof-of-Concept Pilot Study. Journal of Electrical and Computer Engineering. 2011;2011:1–11. doi: 10.1155/2011/681786

[18] Visentin R, Campos-Náñez E, Schiavon M, Lv D, Vettoretti M, Breton M, et al. The UVA/Padova Type 1 Diabetes Simulator Goes From Single Meal to Single Day. Journal of Diabetes Science and Technology. 2018;12(2):273–281. doi: 10.1177/1932296818757747 29451021 PMC5851236

[19] Xie J. Simglucose v0. 2.1; 2018. Available from: https://github.com/jxx123/simglucose.

[20] Fox I, Wiens J. Reinforcement learning for blood glucose control: Challenges and opportunities; 2019. Available from: https://openreview.net/forum?id=ByexVzSAs4.

[21] Lim MH, Lee WH, Jeon B, Kim S. A Blood Glucose Control Framework Based on Reinforcement Learning With Safety and Interpretability: In Silico Validation. IEEE Access. 2021;9:105756–105775. doi: 10.1109/ACCESS.2021.3100007

[22] Sutton RS, Barto AG. Reinforcement learning: An introduction. MIT press; 2018.

[23] Meng L, Gorbet R, Kulić D. Memory-based Deep Reinforcement Learning for POMDP. arXiv preprint arXiv:210212344. 2021;.

[24] Haarnoja T, Zhou A, Abbeel P, Levine S. Soft actor-critic: Off-policy maximum entropy deep reinforcement learning with a stochastic actor. In: International Conference on Machine Learning. PMLR; 2018. p. 1861–1870.

[25] Schulman J, Wolski F, Dhariwal P, Radford A, Klimov O. Proximal policy optimization algorithms. arXiv preprint arXiv:170706347. 2017;.

[26] Diabetes Fact Sheet; 2020. Available from: https://www.who.int/en/news-room/fact-sheets/detail/diabetes.

[27] Diagnosing Diabetes;. Available from: https://dtc.ucsf.edu/types-of-diabetes/type2/understanding-type-2-diabetes/basic-facts/diagnosing-diabetes.

[28] Resalat N, Youssef JE, Tyler N, Castle J, Jacobs PG. A statistical virtual patient population for the glucoregulatory system in type 1 diabetes with integrated exercise model. PLOS ONE. 2019;14(7):e0217301. doi: 10.1371/journal.pone.0217301 31344037 PMC6657828

[29] Salas N, Ferguson B, Zweig J. Reinforcement learning for personalized medication dosing;.

[30] Woldaregay AZ, Årsand E, Walderhaug S, Albers D, Mamykina L, Botsis T, et al. Data-driven modeling and prediction of blood glucose dynamics: Machine learning applications in type 1 diabetes. Artificial Intelligence in Medicine. 2019;98:109–134. doi: 10.1016/j.artmed.2019.07.007 31383477

[31] Brockman G, Cheung V, Pettersson L, Schneider J, Schulman J, Tang J, et al. Openai gym. arXiv preprint arXiv:160601540. 2016;.

[32] Eysenbach B, Levine S. If MaxEnt RL is the answer, what is the question? arXiv preprint arXiv:191001913. 2019;.

[33] Mao H, Venkatakrishnan SB, Schwarzkopf M, Alizadeh M. Variance reduction for reinforcement learning in input-driven environments. arXiv preprint arXiv:180702264. 2018;.

[34] Fujimoto S, Hoof H, Meger D. Addressing function approximation error in actor-critic methods. In: International Conference on Machine Learning. PMLR; 2018. p. 1587–1596.

[35] Zhao W, Chu H, Miao X, Guo L, Shen H, Zhu C, et al. Research on the Multiagent Joint Proximal Policy Optimization Algorithm Controlling Cooperative Fixed-Wing UAV Obstacle Avoidance. Sensors. 2020;20(16):4546. doi: 10.3390/s20164546 32823783 PMC7471982

[36] Trevitt S, Simpson S, Wood A. Artificial Pancreas Device Systems for the Closed-Loop Control of Type 1 Diabetes. Journal of Diabetes Science and Technology. 2016;10(3):714–723. doi: 10.1177/1932296815617968 26589628 PMC5038530

[37] Schoelwer MJ, DeBoer MD. Artificial Pancreas Technology Offers Hope for Childhood Diabetes. Current Nutrition Reports. 2021;10(1):47–57. doi: 10.1007/s13668-020-00347-9 33411096

[38] Nimri R, Grosman B, Roy A, Nir J, Shvalb NF, Kurtz N, et al. Feasibility Study of a Hybrid Closed-Loop System with Automated Insulin Correction Boluses. Diabetes Technology & Therapeutics. 2021;23(4):268–276. doi: 10.1089/dia.2020.0448 33185480

[39] Jalalimanesh A, Haghighi HS, Ahmadi A, Soltani M. Simulation-based optimization of radiotherapy: Agent-based modeling and reinforcement learning. Mathematics and Computers in Simulation. 2017;133:235–248. doi: 10.1016/j.matcom.2016.05.008

[40] Petersen BK, Yang J, Grathwohl WS, Cockrell C, Santiago C, An G, et al. Precision medicine as a control problem: Using simulation and deep reinforcement learning to discover adaptive, personalized multi-cytokine therapy for sepsis. arXiv preprint arXiv:180210440. 2018;.

[41] Paula MD, Ávila LO, Martínez EC. Controlling blood glucose variability under uncertainty using reinforcement learning and Gaussian processes. Applied Soft Computing. 2015;35:310–332. doi: 10.1016/j.asoc.2015.06.041

[42] Kovatchev BP, Breton M, Man CD, Cobelli C. In SilicoPreclinical Trials: A Proof of Concept in Closed-Loop Control of Type 1 Diabetes. Journal of Diabetes Science and Technology. 2009;3(1):44–55. doi: 10.1177/193229680900300106 19444330 PMC2681269

[43] Hausknecht M, Stone P. Deep recurrent q-learning for partially observable mdps. arXiv preprint arXiv:150706527. 2015;.

[44] Caccomo S. FDA approves automated insulin delivery and monitoring system for use in younger pediatric patients. The United States Food and Drug Administration. 2018.

[45] Clarke W, Kovatchev B. Statistical Tools to Analyze Continuous Glucose Monitor Data. Diabetes Technology & Therapeutics. 2009;11(S1):S-45–S-54. doi: 10.1089/dia.2008.0138 19469677 PMC2903980

[46] Wei T, Webb B. A Bio-inspired Reinforcement Learning Rule to Optimise Dynamical Neural Networks for Robot Control. In: 2018 IEEE/RSJ International Conference on Intelligent Robots and Systems (IROS). IEEE; 2018. p. 556–561.

[47] Battelino T, Danne T, Bergenstal RM, Amiel SA, Beck R, Biester T, et al. Clinical Targets for Continuous Glucose Monitoring Data Interpretation: Recommendations From the International Consensus on Time in Range. Diabetes Care. 2019;42(8):1593–1603. doi: 10.2337/dci19-0028 31177185 PMC6973648

[48] Xie J. How did you obtain the parameters in vpatient_params.CSV?;. Available from: https://github.com/jxx123/simglucose/issues/26.

[49] Pompa M, Panunzi S, Borri A, De Gaetano A. A comparison among three maximal mathematical models of the glucose-insulin system. PLoS One. 2021;16(9):e0257789. doi: 10.1371/journal.pone.0257789 34570804 PMC8476045

[50] Raffin A, Hill A, Ernestus M, Gleave A, Kanervisto A, Dormann N. Stable Baselines3; 2019. Available from: https://github.com/DLR-RM/stable-baselines3.

[51] Guadarrama S, Korattikara A, Ramirez O, Castro P, Holly E, Fishman S, et al. TF-Agents: A library for reinforcement learning in tensorflow; 2018. Available from: https://www.tensorflow.org/agents.

[52] Holzleitner M, Gruber L, Arjona-Medina J, Brandstetter J, Hochreiter S. Convergence Proof for Actor-Critic Methods Applied to PPO and RUDDER. In: Transactions on Large-Scale Data- and Knowledge-Centered Systems XLVIII. Springer Berlin Heidelberg; 2021. p. 105–130.

[53] Zhao W, Queralta JP, Westerlund T. Sim-to-Real Transfer in Deep Reinforcement Learning for Robotics: a Survey. In: 2020 IEEE Symposium Series on Computational Intelligence (SSCI). IEEE; 2020. p. 737–744.

[54] Levine S, Kumar A, Tucker G, Fu J. Offline Reinforcement Learning: Tutorial, Review, and Perspectives on Open Problems; 2020. arXiv preprint arXiv:2005.01643. 2020;.

[55] Janner M, Li Q, Levine S. Offline Reinforcement Learning as One Big Sequence Modeling Problem. In: Advances in Neural Information Processing Systems; 2021. p. 1–14.

[56] Chen L, Lu K, Rajeswaran A, Lee K, Grover A, Laskin M, et al. Decision Transformer: Reinforcement Learning via Sequence Modeling. In: Beygelzimer A, Dauphin Y, Liang P, Vaughan JW, editors. Advances in Neural Information Processing Systems; 2021. p. 1–14. Available from: https://openreview.net/forum?id=a7APmM4B9d.

